# Sexually dimorphic and clock gene-specific effects of artificial light at night on *Drosophila* behavioural rhythms

**DOI:** 10.1098/rspb.2025.1170

**Published:** 2025-08-27

**Authors:** Ausra Pranevicius, Grace Biondi, Aishwarya Ramakrishnan Iyer, Araceli Seiffe, Amaicha Mara Depino, Maria de la Paz Fernandez

**Affiliations:** ^1^ Neuroscience and Behavior, Barnard College, New York, NY 10027, USA; ^2^ Department of Biology, Indiana University, Bloomington, IN 47405, USA; ^3^ Institute of Psychiatry and Neuroscience of Paris, 75014 Paris, France; ^4^ Departamento de Biodiversidad y Biología Experimental, Universidad de Buenos Aires Facultad de Ciencias Exactas y Naturales, Buenos Aires C1428, Argentina; ^5^ IFIBYNE, Buenos Aires C1428EGA, Argentina

**Keywords:** behavioural genetics, sex differences, *Drosophila*, circadian rhythms, clock genes, artificial light at night

## Abstract

Light pollution is a major anthropogenic environmental change and a significant threat to ecosystems. Among other detrimental effects on physiology, artificial light at night (ALAN) disrupts circadian rhythms in a wide range of species. However, the underlying neuronal and genetic mechanisms remain poorly understood. Here, we show in *Drosophila* that the loss of the circadian clock gene *period* exacerbates the ALAN-induced shift towards nocturnal behaviour, with a more pronounced effect on males. In contrast, the loss of *cycle* has no such effect on males or females; *cyc* null mutants are nocturnal under standard light‒dark cycles, and their activity and sleep profiles are minimally or not affected by ALAN exposure. CRISPR-Cas9 knockout of *period* in most clock neurons resembles the null mutant phenotype. Our results show that mutations in components of the positive and negative limbs of the circadian clock result in distinct responses to nocturnal light and highlight the role of genetic factors in modulating behavioural plasticity in response to environmental perturbations.

## 1. Introduction

Light pollution is one of the main anthropogenic changes to the environment and is an increasing threat to ecosystems [1,2]. Artificial light at night (ALAN) represents a threat to pollination [3], can disrupt competitive interactions [4] and predator‒prey relationships [5], and has profound effects on nocturnal insect species [6]. The last century has seen a clear increase in the use of light at night; over the past 12 years, the increase has been 12% annually [7]. Most of the human population currently lives in environments that experience some form of ALAN [8]. The natural daily cycles of bright days and dark nights orchestrate metabolic, physiological and behavioural rhythms, and among other detrimental effects on physiology, circadian rhythms and sleep patterns are disrupted by light pollution [9–13].

Circadian clocks, present in nearly all organisms, evolved as a mechanism to maintain internal temporal order and anticipate daily environmental changes [14,15], and their disruption has negative effects on health [16,17]. The *Drosophila melanogaster* timekeeping system, comparable to the mammalian suprachiasmatic nuclei (SCN), comprises approximately 240 neurons that display synchronous oscillation in the abundance of the clock genes *period* (*per*) and *timeless* (*tim*) (reviewed in [18,19]). Clock neurons can be divided into distinct neuronal populations based on gene expression, anatomy, location in the brain, and connectivity patterns [20–25]. The small ventral lateral neurons (s-LN_v_s) that release the neuropeptide pigment dispersing factor (PDF) [26,27] are considered the most dominant pacemakers within the clock neuron network since they are key for maintaining rhythmicity under constant temperature and darkness (DD) [21,28,29].

Under standard (LD) conditions consisting of 12 h of bright light followed by 12 h of darkness, *Drosophila* exhibit a bimodal locomotor activity pattern with peaks occurring near dawn and dusk. These activity peaks are preceded by gradual increases in activity, a feature of a functional circadian clock. Under these conditions, females display higher levels of daytime activity and reduced daytime sleep compared with males [30–33]. When wild-type males are exposed to dim nocturnal illumination (approx. 0.01 lux, comparable to moonlight), the morning activity peak advances by approximately 1 h, whereas the evening peak is delayed by 3 h relative to LD conditions [34]. In these flies, the molecular circadian clock is also altered: the phase of clock protein oscillations is advanced in the s-LN_v_s, which mediate morning activity, and delayed in the lateral dorsal neurons (LN_d_s), which are associated with evening activity [34].

Studies using brighter nocturnal light intensities that mimic urban light pollution (1–10 lux) have also shown that ALAN decreases female fecundity and adult survival [35] and reduces the levels of reactive oxygen species [36]. Additionally, flies exposed to ALAN appear more vulnerable to metabolic stress [36]. Here, we show that ALAN alters *Drosophila* sleep/wake cycles by increasing nocturnal activity, with a greater impact on males than on females. ALAN exposure dampens molecular oscillations in s-LN_v_s, suggesting that other clock neuron groups can sustain behavioural rhythms under these conditions. We found that a null mutation in *per*, a core component of the circadian molecular feedback loop, increased the behavioural response to ALAN, particularly in males. Notably, *cycle* (*cyc^01^*) mutants, which are already nocturnal under LD, do not exhibit behavioural changes under ALAN. A CRISPR-Cas9-mediated *per* knockout restricted to clock neurons mimics the phenotype of *per* null mutants. These findings show that ALAN disrupts *Drosophila* activity rhythms in a sex- and genotype-specific manner and reveal that distinct core clock gene mutations produce differential behavioural responses to nocturnal light.

## 2. Material and methods

### (a) Fly lines and rearing

Flies were raised on cornmeal–sucrose yeast medium in a Percival incubator at 25°C under 12 h light (500 lux)c : 12 h dark (0 lux) cycles (LD). Information about the genotypes and identifier numbers for all the genotypes can be found in table 1. The line SS36676 from the Janelia splitGal4 collection used to label the E3 cluster of clock neurons (PDFR/CRY-negative LN_d_s) was generated by Aljoscha Nern (Janelia Research Campus) in collaboration with the Janelia Fly Light Project Team [41].

**Table 1.** Reagents and resources. PBS, phosphate-buffered saline.

reagent or resource	source	identifier
*fly strain*		
*w;Clk856-Gal4*	Bloomington *Drosophila* Stock Center	BDSC 93198
*per^01^*	Bloomington *Drosophila* Stock Center	BDSC 80928
*w^1118^*	Bloomington *Drosophila* Stock Center	BDSC 5905
*Canton-S*	Bloomington *Drosophila* Stock Center	BDSC 64349
*cyc^01^*	Bloomington *Drosophila* Stock Center	BDSC 80929
*w;UAS-per-gs;UAS-Cas9*	M. Rosbash, Brandeis	[37]
*E3* Split Gal4 (SS36676) (*w*; VT040042-p65ADZp in attP40; R31C03-GAL4.DBD in attP2)	Janelia Split Gal4 collection; donated by Aljoscha Nern, Janelia Research Campus	BDSC 88484
*y*^1^*w*;pin/Cyo; uas-mCD8-GFP	Bloomington *Drosophila* Stock Center	BDSC 5130
*antibody*		
mouse anti-PDF (1 : 3000 or 1 : 2000)	Developmental Studies Hybridoma Bank	PDF C7
rabbit anti-GFP (1 : 2000)	Thermo Fisher	A-11122
rat anti-PER (1 : 500)	Orie Shafer (IU Bloomington)	
chicken anti-GFP (1 : 2000)	Rockland	600-901-215
anti-mouse Alexa-488 (1 : 3000)	Thermo Fisher	A11029
anti-rabbit Alexa-488 (1 : 3000)	Thermo Fisher	A48282
anti-rat Alexa-568 (1 : 3000)	Thermo Fisher	A78946
anti-rabbit Alexa-568 (1 : 3000)	Thermo Fisher	A11036
anti-chicken Alexa-488 (1 : 3000)	Thermo Fisher	A11039
*software*		
Fiji	http://fiji.sc	RRID: SCR_002285
PHASE	open-source software: https://github.com/ajlopatkin/PHASE	[38]
MATLAB R2022b	MathWorks, Natick, MA	RRID: SCR_001622
GraphPad Prism 10.4.0	GraphPad software	RRID: SCR_002798
DAM FileScan	Trikinetics	
R nlme package	https://cran.r-project.org/web/packages/nlme/index.html	
R lmerTest package	https://cran.r-project.org/web/packages/lmerTest/index.html	[39]
R glmmTMB package	https://cran.r-project.org/web/packages/glmmTMB/index.html	[40]
*chemical*		
Vectashield mounting medium	Vector Laboratorie	H-1000-10
premix PBS buffer (10×)	Sigma-Aldrich	11666789001
2% paraformaldehyde (PFA)	Sigma-Aldrich	47608-250ML-F
Triton X-100	Bio Basic	CAS no. 9002-93-1
Schneider’s *Drosophila* medium	Thermo Fisher	2 17 20 024

### (b) Immunohistochemistry

Brains of 6−8 day old adult males were dissected at specified timepoints (ZT02, ZT06, ZT10, ZT14, ZT18 and ZT22) in ice-cold Schneider’s *Drosophila* Medium (S2) (Thermo Fisher, no. 21720024). They were fixed immediately after dissection in 2% paraformaldehyde (PFA) in S2 for 30 min. Brains were then treated with blocking solution, 5% goat serum in phosphate-buffered saline (PBS) + 0.3% Triton (PBS-Tx), for 1 h at 25°C, followed by incubation with primary antibodies at 4°C for 24−48 h. After incubation, brains were rinsed six times in 0.3% PBS-Tx, after which they were incubated with Alexa Fluor-conjugated secondary antibodies for 24 h at 4°C. A list of all antibodies used can be found in table 1. The brains were washed six times with 0.3% PBS-Tx and mounted on a clean glass slide in Vectashield (Vector Laboratories, no. H-1000-10) mounting medium.

### (c) Imaging, quantification and statistical analysis

All images were acquired on an Olympus Fluoview FV3000 laser-scanning confocal microscope (Olympus, Center Valley, PA). Only one hemisphere per brain was imaged. Single optical sections of clock neurons were imaged via the same settings with a 40×/1.10 objective. PER levels were determined through normalization of nuclear staining within each cell to the background signal using Fiji (ImageJ). The average intensity value for each brain within a clock neuron group (s-LN_v_s or E3) was computed by averaging the values obtained from multiple cells within that group. The s-LN_v_s were identified through co-staining with anti-PDF, while the E3 cells were identified by green fluorescent protein (GFP) expression under a specific split-Gal4 driver (no. SS36676). Quantification was conducted via images from 6 to 12 brains per timepoint. Data were analysed using a two-way ANOVA in R.

### (d) Locomotor activity and sleep recording and analysis

#### (i) Recording

DAM2 *Drosophila* Activity Monitors (TriKinetics, Waltham, MA) were used to record the locomotor activity rhythms of adult male and virgin female flies aged 3–5 days, as previously described [42]. Flies were entrained to 12 : 12 LD or ALAN cycles for at least 5 days and then transferred to constant darkness (DD) for at least 8 days at a constant temperature of 25°C.

#### (ii) Locomotor activity analysis

Activity counts were collected at 1 min intervals, which were subsequently binned into 30 min intervals for conducting time series analysis of locomotor activity. Averaged population activity profiles of specific genotypes in entrainment (LD or ALAN) were generated in MATLAB (MathWorks, Natick, MA) Avia PHASE [38]. First, individual fly activity levels were normalized by establishing the average activity across all 30 min bins over days 2−4 under LD or ALAN as 1.0. Subsequently, population averages of this normalized activity were computed for each 30 min bin. Finally, the population averages for LD or ALAN cycles were averaged into a single representative 24 h day.

#### (iii) Phase analysis

Morning and evening phases were quantified in PHASE using a peak distance (frame length) of 241 min and a filter order of 3. The peak distance setting determines whether PHASE includes ‘shoulder’ peaks as part of a single activity peak or treats them as distinct events. A Savitzky−Golay filter was applied to smooth the activity data, enabling unbiased phase detection relative to specific zeitgeber times (ZTs). In this study, ZT0 was used for the morning phase and ZT12 for the evening phase (for an example of phase values for individual flies, see electronic supplementary material, figure S2*a*).

#### (iv) Sleep analysis

Individual fly sleep minutes were normalized across all 30 min bins over days 2−4 under LD or ALAN by classifying 5 min periods of inactivity as sleep [43]. Population averages of this normalized activity were computed for each 30 min bin and averaged into a single representative 24 h day. Total daytime (ZT0−12), nighttime (ZT12−24), morning (ZT0−3) and evening (ZT9−12) sleep counts from days 2 to 4 of LD were recorded. Population sleep averages for LD or ALAN cycles were averaged into a single representative 24 h day.

### (e) Statistical analysis

We used R [44] for the statistical analysis, using the nlme package [45] to fit a linear model via generalized least squares. In cases where the assumption of homoscedasticity was not met, the variance was modelled. When analysing sleep time, the lmerTest package [39] was used to fit linear mixed-effects models for each variable, with each fly as a random factor. Full sets of comparisons were only performed when we observed an interaction among all factors, and in such cases, *post hoc* comparisons were performed with the Tukey test via the emmeans package [46] and asterisks were used to indicate differences. In cases with only main factor effects (e.g. sex, environment or genotype), joint brackets and the numeral symbol were used to indicate the significant effects. When the data did not fit a normal distribution, we used the glmmTMB package [40] to fit generalized linear mixed-effects models (GLMMs). The specific analysis used in each case is indicated in the figure legend. In all the cases, statistical significance was assumed when *p* < 0.05.

## 3. Results

### (a) Nocturnal light has sexually dimorphic effects on activity and sleep

**Figure 1.**
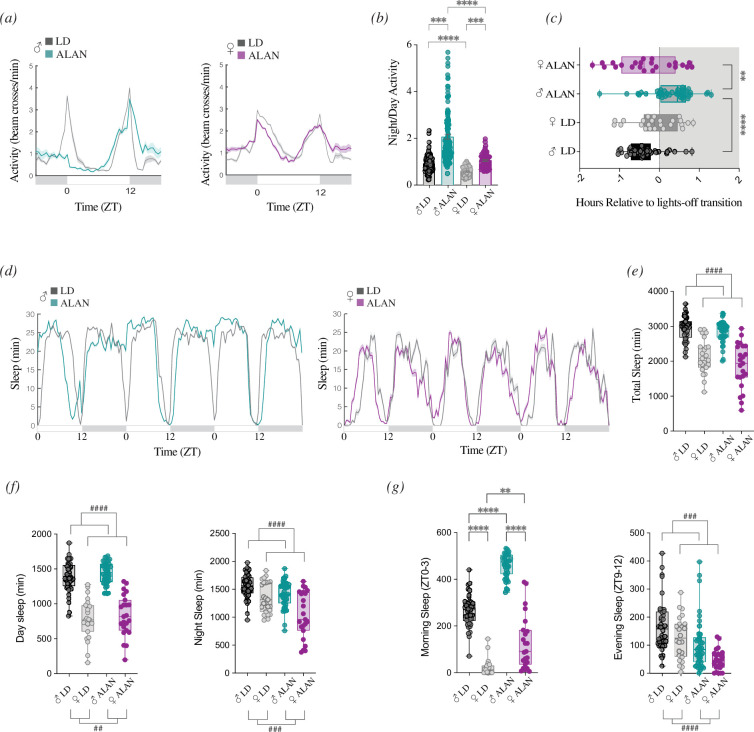
Behavioural responses to light at night (ALAN) are sexually dimorphic. (*a*) Population activity plots of male (left) and female (right) *w^1118^* flies averaged over 3 days under standard light–dark (LD; grey) or ALAN (10 lux) conditions at 25°C. Male activity is shown in green, and female activity is shown in magenta. (*b*) Nocturnality ratios (night activity/total activity) were quantified in males and females under LD and ALAN. (*c*) The timing of the evening activity peak measured relative to lights-off (ZT12) compared across conditions. (*d*) Population sleep plots of male (left) and female (right) flies under LD and ALAN conditions, averaged across 3 days. (*e*) Total minutes of sleep duration were measured under each condition. Sleep was defined as 5 min of inactivity. (*f,g*) Quantification of sleep minutes on days 2−4 during (*f*) daytime (ZT0−12) and nighttime (ZT12−24), and (*g*) morning (ZT0−3) and evening (ZT09−12). Statistical analyses were performed using generalized least squares (GLS) models. Full sets of comparisons were only performed when we observed an interaction among all factors, and in such cases, *post hoc* pairwise comparisons were conducted via estimated marginal means (EMMs) and are indicated with asterisks. In cases with only main factor effects (e.g. sex and/or environment), joint brackets and the numeral symbol are used to indicate the significant effects. Error bars indicate s.e.m. Each dot represents one fly. Only asterisks that correspond to relevant comparisons are shown. *p < 0.05, **p < 0.01, ***p < 0.001, ****p < 0.0001.

To determine the effects of light at night on sleep/wake cycles, we exposed adult flies to 5 days of 12 : 12 light‒dark cycles consisting of standard bright days (500 lux) and either a completely dark night (LD) or a dimly illuminated night (10 lux, ALAN). Similarly to what was reported for males exposed to moonlight-like conditions (0.01 lux) [34], we observed an increase in nocturnal activity (figure 1*a*,*b*). This effect was more pronounced in males than in females. The male nocturnality ratio was 2.22-fold greater under ALAN than under LD, whereas the female ratio increased by 1.75-fold under ALAN (figure 1*b*). The increase in nocturnal activity in males was most evident at the beginning of the night. Males exhibited a significant delay in the phase of the evening peak of activity, likely owing to the increased activity after the lights-off transition (figure 1*c*; electronic supplementary material, figure S1*a*). Interestingly, males but not females showed a suppression of activity in the morning when transitioning from a dimly illuminated night to bright light (ZT0 under ALAN; figure 1*a*). ALAN did not affect the phase of the morning activity peak in either sex (electronic supplementary material, figure S1*b*).

We next quantified sleep levels under each environmental condition [43]. Consistent with reduced male activity after lights-on, morning sleep (ZT0−3) was increased in males under ALAN (figure 1*d*, left). However, total sleep levels were unaffected by light condition, with a main effect of sex but not environment (figure 1*e*). As previously reported under LD, females showed reduced daytime sleep compared with males (ZT0−12), and this pattern persisted under ALAN (figure 1*f*). ALAN also induced a pronounced increase in male morning sleep (ZT0−3) and a reduction in evening sleep (ZT9−12) in both sexes (figure 1*g*). Daytime bout length was shorter in females under both conditions, whereas at night it was shorter only in males (electronic supplementary material, figure S1*c*). ALAN reduced daytime sleep latency in both sexes, while nighttime latency increased under ALAN regardless of sex (electronic supplementary material, figure S1*d*).

After 5 days under either LD or ALAN, flies were transferred to constant darkness (DD) to evaluate the impacts of ALAN exposure (electronic supplementary material, figure S2*a*,*b*). Males exposed to standard light‒dark cycles slept significantly less on the first subjective day (CT0−12) than during the daytime on the last day of LD (ZT0−12) (electronic supplementary material, figure S2*b*, top left). In contrast, males exposed to ALAN increased their sleep during the first subjective day compared with daytime sleep levels during the last day of entrainment (electronic supplementary material, figure S2*b*, bottom left). Females showed similar phenotypes, although the effects were less pronounced (electronic supplementary material, figure S2*b,* right). Taken together, these results show that male and female sleep are differentially affected by nocturnal light exposure.

### (b) Subcellular localization of circadian clock proteins is impaired by nocturnal light

**Figure 2.**
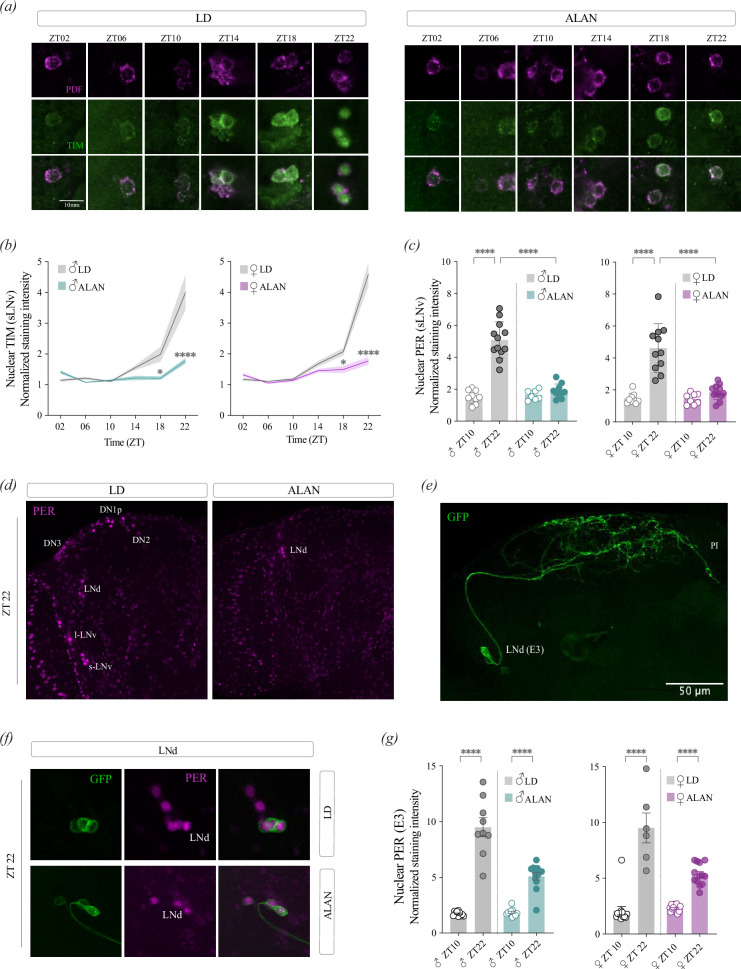
Artificial light at night (ALAN) disrupts nuclear accumulation of circadian clock proteins in clock neurons. (*a*) Representative confocal images of TIM staining in the small ventral lateral neurons (s-LN_v_s) at the indicated timepoints under light-dark (LD) or ALAN conditions. Pigment dispersing factor (PDF; magenta) marks s-LN_v_s and delimits cytoplasmic boundaries. Scale bar, 10 mm. (*b*) Quantification of nuclear TIM intensity in s-LN_v_s across timepoints in males (left) and females (right) under LD (grey) or ALAN (males, green; females, magenta). (*c*) Quantification of nuclear PERIOD (PER) levels in s-LN_v_s at two antiphasic timepoints (ZT10 and ZT22) in males (left) and females (right) under LD or ALAN. (*d*) Representative confocal images showing PER expression at ZT23 under LD (left) and ALAN (right) conditions. (*e*) Representative expression of the E3-specific split-Gal4 line (*SS36676 > mCD8*-*GFP*), showing expression in the LN_d_ cluster. Scale bar, 50 µm. (*f*) Confocal images of PER (magenta) in *SS36676 > mCD8*-*GFP* flies at ZT23 under LD (top) or ALAN (bottom) conditions. (*g*) Quantification of nuclear PER intensity in E3 neurons at ZT10 and ZT22 in males (left) and females (right) under LD or ALAN. Error bars indicate s.e.m. Each dot represents one fly. Statistical comparisons were performed using two-way ANOVA with Tukey’s multiple comparisons test. Only asterisks that correspond to relevant comparisons are shown. *p < 0.05, ****p < 0.0001.

Moonlight exposure shifts the phase of the clock in *Drosophila* males, resulting in phase advance in some clock neuron groups and phase delays in others [34]. However, the light intensity of ALAN conditions is higher than moonlight conditions. TIM is light sensitive, and PER becomes unstable without TIM (reviewed in [47]). We analysed the subcellular localization of circadian clock proteins in the s-LN_v_s and found that while TIM accumulates in the nucleus towards the end of the night (ZT22) under LD conditions as expected, while it remains low and predominantly cytoplasmic under ALAN throughout the night (figure 2*a*,*b*). No differences were found between males and females; the TIM nuclear signal was significantly lower under ALAN than under LD at ZT18 and ZT22 in both males and females (figure 2*b*). A similar pattern was observed for PER when nuclear intensity was at two antiphasic timepoints, ZT10 and ZT22 (figure 2*c*). Overall PER levels were lower under ALAN compared with LD in both males and females (electronic supplementary material, figure S2*c*).

While TIM and PER nuclear signals were largely absent in the s-LN_v_s at the end of the night under ALAN conditions, PER was still detectable in other cells (figure 2*d*). Notably, a subset of LN_d_s retained a robust nuclear PER signal at ZT22 under ALAN (figure 2*d*, right panel). We hypothesized that these neurons correspond to cryptochrome-negative (CRY^−^) LN_d_s, also referred to as ‘E3’ evening neurons [48,49]. To test this, we used a split-Gal4 line that labels these neurons (no. SS36676, figure 2*e*) and co-stained for PER and GFP at the end of the night to compare PER levels under LD and ALAN conditions (figure 2*f*). Under ALAN, nuclear PER was detected only in GFP-positive LN_d_s (figure 2*f*). In contrast to those in the s-LN_v_s (figure 2*c*), PER levels in E3 cells still oscillated under ALAN, although the amplitude was lower than that under LD (figure 2*g*). In males under LD, nuclear PER levels were 5.47-fold higher at ZT22 compared with ZT10; under ALAN, the increase was reduced to 2.73-fold. A similar pattern was observed in females, with a 4.53-fold increase under LD and a 2.31-fold increase under ALAN (figure 2*g*). These results reveal that molecular oscillations in specific groups of clock neurons are differentially affected by nocturnal light.

### (c) Clock gene mutants are differentially affected by artificial light at night

While null mutations in any of the core circadian clock genes result in arrhythmicity under free-running conditions, different mutations have distinct effects on behaviour under LD cycles. Null mutations in the genes of the negative limb of the clock, *per* and *tim*, lead to activity profiles that resemble those of wild-type flies, with the notable exception that these mutants cannot anticipate the lights-on and lights-off transitions. However, they still display a bimodal activity pattern because of the ‘startle effect’, i.e. a sharp increase in activity following light transitions, a direct response to light that is not governed by the circadian clock. In contrast, *Clk* and *cyc* null mutants predominantly exhibit nocturnal behaviour [50–53].

**Figure 3.**
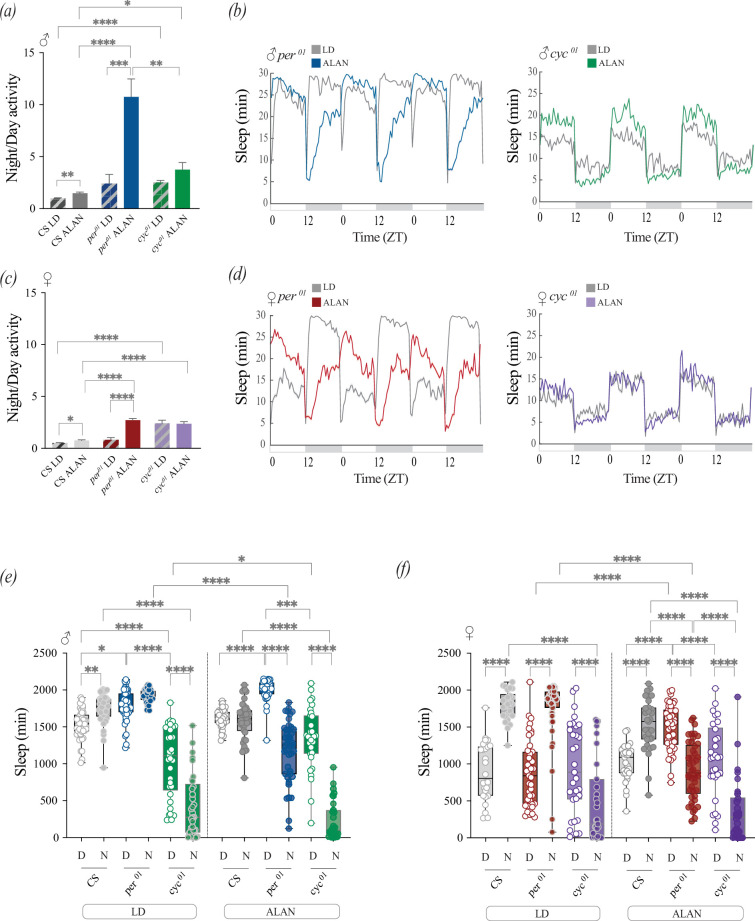
Artificial light at night (ALAN) alters sleep and nocturnality in a genotype- and sex-dependent manner. Sleep data for male and female flies were generated from days 2 to 4 of entrainment under Light-dark (LD) or ALAN. (*a*) Nocturnality ratios were quantified in *Canton-S* (CS; grey), *per^01^* (blue) and *cyc^01^* (green) males under LD (striped) and ALAN (solid) conditions. (*b*) Population sleep plots for male *per^01^* (left) and *cyc^01^* (right) flies under LD (grey) or ALAN (blue and green, respectively) conditions, averaged across days 2−4. (*c*) Nocturnality ratios for female *CS* (grey), *per^01^* (red) and *cyc^01^* (purple) flies under LD (striped) and ALAN (solid) conditions. (*d*) Population sleep plots for female *per^01^* (left) and *cyc^01^* (right) flies under LD (grey) or ALAN (red and purple, respectively), averaged across days 2−4. (*e,f*) Daytime and nighttime sleep durations (min) were averaged over days 2−4 under LD (left) or ALAN (right) conditions for males (*e*) and females (*f*) of the indicated genotypes. Sleep was defined as 5 min of inactivity. Error bars indicate s.e.m. Each dot represents one fly. Statistical analyses were performed using generalized least squares (GLS) models with heterogeneous variance structures for night/day activity data. Full sets of comparisons were only performed when we observed an interaction among all factors, and in such cases *post hoc* pairwise comparisons were conducted via estimated marginal means (EMMs) and are indicated with asterisks. Additionally, mixed models were used for daytime and nighttime sleep analysis, with *post hoc* comparisons also conducted using EMMs. Only asterisks that correspond to relevant comparisons are shown. *p < 0.05, **p < 0.01, ***p < 0.001, ****p < 0.0001.

To determine whether null mutants in different clock components are differentially affected by ALAN, we compared the activity profiles of *per* and *cyc* null mutants with their genetic background control, *Canton-S* (CS). We found that *per^01^* mutant males exhibited an approximately 4.5-fold increase in nocturnality (figure 3*a*; electronic supplementary material, figure S3*a*), a markedly more pronounced effect than that observed in CS males (approx. 1.5-fold). Sleep profiles of *per^01^* males revealed a substantial reduction in nighttime sleep under ALAN conditions (figure 3*a*,*b*). While nighttime and daytime sleep were not significantly different for *per^01^* males under LD, nighttime sleep was approximately 50% lower than daytime sleep when exposed to nocturnal light (figure 3*c*,*e*). In contrast, *cyc^01^* males, which are already nocturnal under standard entrainment conditions, do not show significant changes in nocturnality under ALAN compared with LD (figure 3*a*,*b*,*e*), though they exhibit a mild increase in daytime sleep (figure 3*e*).

Under standard entrainment conditions, females show higher daytime activity and lower daytime sleep compared with males [54–56]. In contrast to Canton-S (CS) males, which do not show increased nighttime sleep under ALAN (figure 3*e*), CS females show greater nighttime sleep under both LD and ALAN conditions (figure 3*f*). However, their nocturnality increases significantly under ALAN (figure 3*c*; electronic supplementary material, figure S3*b*). Compared with control females, *per^01^* females showed a more pronounced increase in nocturnality under ALAN compared with control females (approx. 3.4-fold versus approx. 1.5-fold; figure 3*c*), although this effect was less pronounced than that observed in *per^01^* males. Like control females, *per^01^* females sleep significantly more at night under LD, but exposure to ALAN reversed this pattern, resulting in significantly less nighttime than daytime sleep (figure 3*d*,*f*). *cyc^01^* females did not exhibit any changes in nocturnality or sleep under ALAN conditions compared with LD *(*figure *3d,f*). Compared with those of the controls, total levels of sleep were lower for male and female *cyc* mutants under both conditions (electronic supplementary material, figure S3*c*). These results show that mutations in different components of the molecular clock result in opposite responses to nocturnal light: while the *per* null mutants are more affected than wild-type flies, there is no significant effect on the activity profiles of *cyc* mutants.

### (d) *per* mutagenesis in clock neurons leads to an enhanced response to nocturnal light

**Figure 4.**
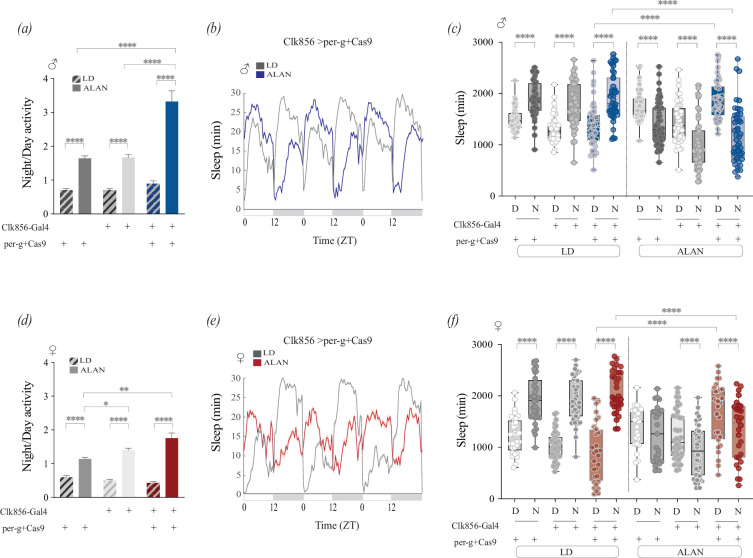
Clock neuron-specific loss of *per* increases sensitivity to artificial light at night (ALAN). CRISPR-Cas9 was used to disrupt *period* expression specifically in clock neurons using the *Clk856-Gal4* driver. Behavioural data from males and females were collected on days 2−4 of entrainment under light-dark (LD) or ALAN conditions at 25°C. (*a–c*) Behavioural responses of males. (*a*) Nocturnality ratios were quantified in parental control and experimental (*Clk856-Gal4 > per-g+Cas9*) males under LD (striped) and ALAN (solid) conditions. (*b*) Population sleep plots for experimental males under LD (grey) and ALAN (blue). (*c*) Day and night sleep duration for *UAS per-g;UAS-Cas9* control (dark grey), *Clk856-Gal4* control (light grey) and experimental (blue) males under LD and ALAN. (*d–f*) Behavioural responses of females. (*d*) Nocturnality ratios for control and experimental females under LD (striped) and ALAN (solid). (*e*) Population sleep plots for experimental females under LD (grey) and ALAN (red). (*f*) Day and night sleep duration for *UAS per-g; UAS-Cas9* control (dark grey), *Clk856-Gal4* control (light grey) and experimental (red) females under LD and ALAN. Each dot represents one fly. Error bars indicate s.e.m. Statistical analyses were performed using generalized least squares (GLS) models with heterogeneous variance structures for night/day activity data. Full sets of comparisons were only performed when we observed an interaction among all factors, and in such cases, *post hoc* pairwise comparisons were conducted via estimated marginal means (EMMs) and indicated with asterisks (see §2). Only asterisks that correspond to relevant comparisons are shown. *p < 0.05, **p < 0.01, ***p < 0.001, ****p < 0.0001.

In addition to clock neurons, *per* is expressed in glia and outside the brain [57–59]. To determine the extent to which the marked increase in nocturnality observed in *per^01^* mutants was due to the loss of *per* expression in clock neurons, we analysed the behavioural responses to light at night in flies in which *per* was knocked out in these cells via CRISPR-Cas9-mediated mutagenesis [37]. Under LD conditions, there were no significant differences in nocturnality between the experimental *Clk856 > per-g+Cas9* males and the control males (figure 4*a*). In contrast, *Clk856 > per-g+Cas9* males showed a more pronounced increase in nocturnality than did the parental controls (figure 4*a*) when exposed to ALAN. The sleep profile of the experimental males resembled that of *per^01^* males. Unlike the parental control lines, *Clk856 > per-g+Cas9* males showed significant increases in daytime sleep and significant decreases in nighttime sleep under ALAN (figure 4*b*,*c*).

*per* mutant females showed a less pronounced increase in nocturnality when exposed to ALAN than males of the same genotype (figure 3*a*,*c*). While *Clk856>per-g+Cas9* males showed significant increases in nocturnality under ALAN, experimental females did not differ from their heterozygote Gal4 parental controls (figure 4*d*). Sleep analyses of *Clk856>per-g+Cas9* females revealed a significant increase in daytime sleep and significant decreases in nighttime sleep under ALAN, similar to the effects observed in males (figure 4*e*,*f*). Total sleep was not affected in experimental males or females (electronic supplementary material, figure S4). Taken together, these results suggest that the loss of *per* expression in clock neurons results in increased sensitivity to nocturnal light in both males and females.

## 4. Discussion

Although the impact of light pollution on biological rhythms has been recognized across taxa, the underlying neuronal and genetic mechanisms remain unclear. We found that nocturnal light induces changes in both activity rhythms and sleep patterns. Both males and females become more nocturnal under ALAN, but males show a greater increase in nocturnality. Males also exhibited delayed evening peaks and suppressed morning activity under ALAN. Previous studies in *Drosophila* have reported increased night activity in males under moonlight conditions or dim nocturnal light [36]. Unlike the morning phase advance observed in males under moonlight conditions, we did not detect a similar effect; however, the nocturnal light intensity used in moonlight experiments was substantially lower (0.03 versus 10 lux). Our data show that ALAN at an ecologically relevant intensity causes sex-dependent alterations in both activity and sleep, indicating dimorphic behavioural plasticity in response to nocturnal light. These effects were not accompanied by overall changes in total sleep, suggesting that ALAN selectively alters the temporal organization of activity rather than global arousal states.

Sex differences in the circadian system and their interactions with environmental conditions have received relatively little attention. Under standard light‒dark cycles, female *Drosophila* have higher daytime activity and less sleep than males, a difference attributed at least in part to the reduced activity of the DN1p group of clock neurons in females [60]. In males, a subset of DN1p clock neurons that promote sleep receives input from male-specific courtship neurons [30,31]. The difference in female and male activity patterns could also be the result of differences in clock neuron activity or sexually dimorphic input pathways, as *Drosophila* males and females differ in both photoreceptor anatomy and light-transduction mechanisms [61]. Sex differences in photoreception have also been reported in the housefly, *Musca domestica*, where males exhibit faster phototransduction kinetics than females do [56]. Anatomical or physiological differences in light input pathways may underlie the sexually dimorphic behavioural responses to ALAN.

Our results show that nocturnal light prevents nuclear accumulation of core circadian repressors within clock neurons. We found that under ALAN, TIM and PER fail to accumulate in the s-LN_v_ nuclei throughout the night. However, PER still localizes to the nuclei of some cells under ALAN, including a subset of lateral neurons (LN_d_s). These cells correspond to CRY-negative and PDFR-negative ‘E3’ evening neurons and may be less sensitive to photic input. We did not detect sex differences in PER oscillations in E3 cells under ALAN, but we cannot rule out the possibility that these or other clock neurons are differentially sensitive to light in males and females. It is possible that E3 cells contribute to behavioural rhythms that persist under ALAN conditions.

A previous study found that under moonlit nights, clock mutants, including *per^01^*, shifted their activity into the night, suggesting that this effect is independent of the clock [62]. Expression of CRY in circadian neurons is necessary for flies to bifurcate their activity bouts under dim light cycles [63]. Under our conditions, where nocturnal light is substantially more intense (10 versus 0.01 lux), *per^01^* mutants show increased sensitivity. In contrast, *cyc^01^* mutants—previously shown to display nocturnality and low sleep levels—were minimally affected by ALAN, suggesting that *cycle* is not required for ALAN-induced behavioural plasticity. *cyc^01^* males showed a mild increase in daytime sleep under ALAN, while females were unaffected. These divergent responses reveal that, while both genes are essential for maintaining free-running rhythms, *per* appears to have a more prominent role in buffering the behavioural consequences of nocturnal light exposure. CRISPR-mediated deletion of *per* in clock neurons partially phenocopied the behavioural effects observed in *per^01^* mutants, indicating that clock neuron-specific expression of *per* contributes to ALAN sensitivity. Together, these results show that light at night alters both molecular and behavioural aspects of the circadian system in *Drosophila*, with effects that are modulated by sex and genetic background.

Disruption of circadian regulation by ALAN is likely to be a general phenomenon across taxa. The core clock genes *period* and *cycle*/*Bmal1* are highly conserved, and ALAN has been shown to alter their expression in mammals, birds and other insects. In rats, exposure to dim light at night significantly reduces the amplitude of *Per1* and *Bmal1* cycling in the SCN [64]. Similar effects have been observed in mice, where dim nocturnal light disrupts circadian clock rhythms in the hypothalamus at both the gene expression and protein levels [65]. In zebra finches, exposure to 5 lux nocturnal light abolishes rhythms in *Clock* and *Cry* and reduces the amplitude of *Per2* cycling, while *Bmal1* cycling amplitude is not affected [66]. In *Parus major*, ALAN alters *Bmal1* expression in the brain, liver, spleen and blood [67]. In mosquitoes, nocturnal light reduces clock gene expression in a sex-specific manner [68,69]. Given the conservation of these pathways, our results may help elucidate general mechanisms through which light pollution affects neural and behavioural rhythms in other species.

## Data Availability

All the data used in this study are publicly available, without restrictions, on Dryad [70]. Supplementary material is available online [71].
